# Correction: Perception of pediatric residents from a tertiary hospital in the city of México regarding their training during the COVID-19 pandemic

**DOI:** 10.1186/s12909-022-03905-7

**Published:** 2022-12-07

**Authors:** Eduardo Bracho Blanchet, Miguel Klünder Klünder, José Antonio Orozco Morales, Carolina Hill De Titto, Diana Avila Montiel

**Affiliations:** 1 Directorate of Research, Hospital Infantil de México Federico Gómez, CDMX México, Calle Dr. Márquez 162, Col. Doctores, CP 06720 Cuauhtémoc, Mexico; 2 Directorate of Education, Hospital Infantil de México Federico Gómez, CDMX México, Calle Dr. Márquez 162, Col. Doctores, CP 06720 Cuauhtémoc, Mexico

**Correction: BMC Med Educ 22, 726 (2022)**

**https://doi.org/10.1186/s12909-022-03776-y**

Following publication of the original article [1], the authors identified an error in the author name of Miguel Klünder Klünder.

The incorrect author name is: Miguel Künder Klünder

The correct author name is: Miguel Klünder Klünder

Also, they informed us that first name and last name of author Eduardo Bracho Blanchet has been incorrectly tagged.

The correct first name is: Eduardo

The correct last name is: Bracho Blanchet

The author group has been updated above and the original article [1] has been corrected.
